# Rural iowa adolescents’ use, knowledge and attitudes regarding UTVs on public roads

**DOI:** 10.1186/s40621-026-00707-z

**Published:** 2026-10-28

**Authors:** Parker R. Sternhagen, Brooke L. Krueger, Cole C. Wymore, Junlin Liao, Pam J. Hoogerwerf, Brenda Vergara, Charles A. Jennissen

**Affiliations:** 1 University of Iowa College of Liberal Arts and Sciences, Iowa City, IA USA; 2 Department of Emergency Medicine, University of Kansas Medical Center, Kansas City, KS USA; 3 Department of Surgery, University of Iowa Healthcare, Iowa City, IA USA; 4 Injury Prevention and Outreach Program, University of Iowa Health Care Stead Family Children’s Hospital, Iowa City, IA USA; 5 Department of Emergency Medicine, Roy J. and Lucille A. Carver College of Medicine, University of Iowa, Iowa City, IA USA; 6 Stead Family Department of Pediatrics, Roy J. and Lucille A. Carver College of Medicine, University of Iowa, Iowa City, IA USA

**Keywords:** Adolescent, Recreational off-highway vehicle, Road, Rural, Side-by-side vehicle, Utility task vehicle, Youth

## Abstract

**Background:**

Manufacturers specify that utility task vehicles (UTVs) are designed exclusively for off-road operation and are unsuitable for public roadways. Despite this, a growing number of U.S. legislative bodies are enacting laws permitting or expanding UTV access to public roads, creating a significant misalignment between vehicle design intent and actual use environments. Our study objective was to examine rural adolescents’ use, knowledge and attitudes regarding UTVs on public roads.

**Methods:**

Iowa FFA (formerly Future Farmers of America) Leadership Conference attendees were anonymously surveyed from April 15–16, 2024. They were questioned about their UTV use and attitudes regarding UTVs on public roadways, both paved and unpaved. Descriptive, bivariate and logistic regression analyses were performed using Stata 15.1.

**Results:**

1,029 FFA members (13–18 yrs) participated; 50% from farms, 20% from the country/not farms, and 30% from towns. 53% of respondents’ families owned UTVs and 69% of respondents had driven/ridden one. Among UTV drivers/riders, 94% had been on unpaved roads (51% at least weekly) and 87% had been on paved roads (41% at least weekly). Overall, 64% and 63% stated it was safe to drive UTVs on unpaved and paved public roads, respectively. 63% stated UTVs were designed for unpaved roads and 54% stated UTVs were designed for paved roads. Over half agreed that manufacturers state UTVs are safe for unpaved (54%) and paved (52%) roads. Generally, males, older teens, UTV owners, UTV riders, those who had ridden on public roads and more frequent riders all had greater proportions holding these misconceptions. The vast majority of respondents (82%) incorrectly believed youth < 18 years old could legally drive UTVs on Iowa’s county roads.

**Conclusions:**

Most study participants reported UTV operation on public roads and perceived the practice as safe on both paved and unpaved roadway surfaces. They also believed the misconceptions that UTVs are designed for roadways and that manufacturers state UTVs are safe on public roads. The powersports industry should do more to publicize the hazards of off-road vehicles on public roads. Targeted education and evidence-based legislation with enforcement prohibiting on-road operation may be critical in decreasing UTV-related deaths and injuries.

**Supplementary Information:**

The online version contains supplementary material available at https://doi.org/10.1186/s40621-026-00707-z.

## Background

Utility Task Vehicles (UTVs) are side-by-side off-road vehicles that have become increasingly popular in recent years for both work and recreation. In fact, the sales of UTVs have more than doubled that of all-terrain vehicles (ATVs) with an estimated 565,000 vehicles sold in North America in 2024 alone [1]. Also known as recreational off-highway vehicles (ROVs), consumers and powersports dealers have more commonly used the term UTV when referring to these vehicles and we will do the same in this report. Although they are both off-road vehicles, UTVs are quite different from ATVs as they have a steering wheel, foot controls for acceleration and braking, and bucket/bench seating. Because UTV’s can travel at least 30 mph (most can reach highway speeds), they are built with a roll-over protective structure (ROPS) and either seatbelts or harness systems to maintain riders within the protective zone afforded by the ROPS.

The increasing number of deaths and non-fatal injuries caused by UTVs has developed into a public health concern. However, as compared to ATVs, there have been relatively few studies specifically investigating UTV-related crashes and injuries [2–9]. In their most recent report published in 2025, the U.S. Consumer Product Safety Commission reported 849 ROV/UTV fatalities from 2019 to 2021, and an estimated 50,700 emergency department-treated injuries from 2019 to 2023 [10]. Studies have shown that a significant proportion of UTV-related crashes have occurred on public highways, estimates ranging from 29 to 61% [3–5, 9]. A study of UTV crashes in the state of Iowa between 2002 and 2019 found that over three-fifths (61%) of UTV-related crashes where location could be determined were on public roadways, and the roadway crashes had more severe outcomes than those off-road [9].

Similar to ATVs, UTVs are designed for off-road use only. Although the risk of a crash with another vehicle is significantly higher for UTVs on public roads than off-road, most public roadway crashes are single vehicle crashes not involving another motor vehicle. Studies have shown that 76–82% of roadway crashes with UTVs are single vehicle crashes not involving another motor vehicle [3, 4, 9]. The Recreational Off-Highway Vehicle Association strongly opposes allowing ROVs/UTVs on public roadways and states that this practice is not safe [11]. UTV manufacturers warn consumers with vehicle stickers and in owner manuals that their vehicles should not be driven on public roadways. In fact, UTVs do not meet Federal Motor Vehicle Safety Standards. They have off-road tires not approved for roadway use that can have unpredictable interactions with public roadway surfaces, both paved and unpaved. In addition, UTVs have a relatively narrow track and high center of gravity making them prone to rollovers, especially at speeds driven on public roads.

An increasing number of cities, counties and states across the U.S. are passing legislation allowing the use of off-highway vehicles (UTVs and ATVs) on public roadways for both transportation and recreational purposes [12–14]. In the past, Iowa allowed counties to individually pass ordinances allowing the use of ATVs/UTVs on their roads. A study examining data through the year 2018 found counties that had passed laws allowing off-road vehicles on public roads had a nearly 60% greater crash rate as compared to before the ordinance went into effect and as compared to counties that had never implemented a law [12]. Despite this, the Iowa legislature passed a law in 2022 allowing the use of UTVs and ATVs on county roads by drivers 18 years and older across the state [15, 16].

Public roadway use is one of the greatest risk factors for UTV crashes. Determining public attitudes and knowledge of this risk, especially in rural areas where UTV use is prevalent, is crucial for improving safety education and developing injury prevention strategies [17]. Our objective was to determine the family ownership and use of UTVs by rural adolescents, especially on unpaved and paved public roads, as well as teenagers’ attitudes and knowledge about safety, design, and manufacturer’s recommendations regarding the use of these vehicles.

## Methods

### Study population

A convenience sample of FFA members were surveyed at the Iowa FFA Leadership Conference on April 15–16, 2024, in Ames, Iowa. FFA is a national high school organization for students interested in agriculture and leadership. Iowa has over 20,000 members in 273 student chapters and has an annual state conference each spring [18]. A total of 4,517 FFA members from 269 chapters attended the conference including 1811 (40%) males, 2655 (59%) females and 51 (1%) with sex/gender undisclosed. Staff and volunteers at the University of Iowa Stead Family Children’s Hospital safety booth invited attendees to complete an anonymous survey on paper or digitally on their phone using a QR code linked to the survey in Qualtrics. Those who completed the survey could play Plinko to win a small prize as an incentive for participating.

### Survey

The survey was created by members of the University of Iowa Stead Family Children’s Hospital (SFCH) Injury Prevention and Outreach Program and in partnership with community stakeholders who are members of the SFCH Off-Road Vehicle Safety Task Force. The goal was to collect data to inform rural adolescent off-road vehicle safety initiatives. For validation purposes, the survey was piloted among twelve adolescents 13–22 years of age in March 2024 prior to the state conference. The survey was provided in written form and participating youth provided oral feedback with discussion regarding the survey to task force members allowing improvements that helped ensure survey clarity.

Demographic variables included age, sex, residence (on a farm, in the country but not on a farm, in town), and race/ethnicity. All were self-identified. A picture and description of UTVs were shown to orient survey takers regarding the vehicle for which they were answering questions. Descriptions of unpaved and paved public roads were also provided to clarify questions regarding roadway surfaces: “An unpaved public road includes gravel or dirt. A paved public road includes concrete, chip and seal, or tar.” Supplementary material includes Additional File 1 which is the UTV survey used in the study.

Respondents were first asked whether they or their family owned a UTV, and then about their UTV usage. Participants were asked how often they drive/ride a UTV in general, on unpaved public roads, and on paved public roads. Responses included: almost daily, about weekly, about monthly, few times a year or less, or never.

Attendees were then asked how safe they felt it was to drive a UTV on an unpaved and on a paved public road, with the response options of very safe, safe, neither safe or unsafe, unsafe, or very unsafe. All survey questions about roadway surfaces were broken into two separate questions, one about unpaved, and the other about paved public roads. Next, respondents were asked to indicate how much they agreed or disagreed with the following statements: (1) UTVs are designed for unpaved/paved public road surfaces; and (2) UTV manufacturers state UTVs are safe to ride on unpaved/paved public roads. Respondents could answer with strongly agree, agree, neither agree or disagree, disagree, or strongly disagree.

Lastly, respondents were asked two questions regarding ATV/UTV laws in Iowa. They were queried: (1) Were you aware that the Iowa Legislature passed a law in 2022 allowing ATVs and UTVs on all unpaved county roads and with some restrictions on two-lane state and county paved highways? (Yes, No) and (2) If you have a valid driver’s license, how old do you need to be to drive ATVs and UTVs on county roads? (14 years, 15 years, 16 years, 17 years, 18 years).

### Data analysis

The University of Iowa SFCH Injury Prevention and Outreach program provided the completed surveys to the research team for study and analysis. Paper surveys were entered into Qualtrics and compiled with those entered digitally by mobile devices. An Excel spreadsheet of the data was then exported into the software program Stata 15.1 (StataCorp, College Station, Texas) for analysis. While all conference attendees were invited to participate in the survey, the study sample was limited to those 13–18 years of age. Only those who could not read the survey were excluded from participating; although, we are unaware of anyone who was not able to participate because of this reason.

Descriptive and bivariate analyses were performed. In addition, multivariable logistic regression models were used to examine factors associated with the perceived safety of UTV use on public roads. Model results are presented as adjusted odds ratios (aORs) with 95% confidence intervals. Predictor variables were selected a priori based on prior literature and study objectives and included sex, age group, place of residence, UTV ownership, and prior UTV roadway use. The primary outcomes examined were perceptions on the safety of UTV use on unpaved and paved public roads.

The eight individuals that identified as non-binary were excluded from comparative analyses by sex due to the low frequency. For comparative analyses by age, the ages were categorized as younger teens (13–15 years) and older teens (16–18 years). Additionally, comparisons by race/ethnicity were not performed due to the lack of racial and ethnic diversity in the sample.

Missing data were handled using available-case analysis, meaning that each specific analysis included only participants with valid data for the variables under consideration. This was deemed appropriate as the proportion of missing responses ranged from 0% to 5.6% for variable/survey questions. All p-values were two-tailed and a value of < 0.05 was considered statistically significant. Given the exploratory nature of the analyses and the use of a convenience sample, results should be interpreted with emphasis on the magnitude and direction of associations rather than statistical significance alone. As the study involved the use of pre-existing, anonymous data, the Institutional Review Board deemed the study exempt.

## Results

### Demographics

A total of 1,029 FFA members completed the survey which was 23% of all conference attendees. However, we estimate that at least 85% of the attendees who were approached to participate did so. Females were 60% of the respondents which was consistent with the majority of conference attendees being female. See Table 1. About two-thirds of participants (66%) were between 16 and 18 years of age; 34% were 13–15 years. Half (50%) lived on a farm, 20% lived in the country but not on a farm, and 30% lived in towns. The vast majority (96%) were of non-Hispanic White race/ethnicity.

**Table 1:** Demographics and UTV use among 2024 Iowa FFA Leadership Conference survey participants (*N* = 1,029)

	*n* (Col %)^a^
Sex	
Female Male Non-binary	605 (59) 416 (40) 8 (1)
Age Grouping	
13–15 years 16–18 years	403 (39) 626 (61)
Where Respondents Live	
Farm Country/Not a Farm Town	512 (50) 209 (20) 308 (30)
Race/Ethnicity	
White/Caucasian Hispanic or Latinx Asian Black/African American Other/Mixed	987 (96) 19 (2) 8 (1) 5 (0) 9 (1)
Own Vehicle	
Yes No	476 (47) 533 (53)
Ridden a UTV	
Yes No	700 (69) 310 (31)
Frequency of UTV Riding^b^	
Almost Daily About Weekly About Monthly Few Times a Year	215 (31) 182 (26) 125 (18) 178 (25)
Driven/Ridden UTV on Unpaved Public Road^b^	
Yes No	652 (94) 41 (6)
UTV Frequency on Unpaved Public Road^b^	
Almost Daily About Weekly About Monthly Few Times a Year Never	187 (27) 168 (24) 126 (18) 171 (25) 41 (6)
Driven/Ridden UTV on Paved Public Road^b^	
Yes No	606 (87) 87 (13)
UTV Frequency on Paved Public Road^b^	
Almost Daily About Weekly About Monthly Few Times a Year Never	129 (19) 155 (22) 137 (20) 185 (27) 85 (13)

^a^The sum of n may not equal the total group N due to missing values, and percentages may not add up to 100 due to rounding

^b^Only includes those who had driven/ridden a UTV in past year

### UTV usage

About half of attendees (47%) stated their family owned at least one UTV. See Table 1. Over two-thirds (69%) of respondents had driven/ridden a UTV in the past year with 57% of these stating they rode a UTV at least weekly. Of FFA members that had driven/ridden a UTV in the past year, 94% had been on an unpaved public road with about half (51%) stating they did so at least weekly. In addition, 87% had driven/ridden a UTV on a paved public road in the past year with over two-fifths (41%) doing so at least weekly. Of drivers/riders overall, 96% (656/686) had driven/ridden UTVs on a public road in the past year.

### UTV driving/riding comparisons by sex, age and residence

A higher proportion of males stated their families owned at least one UTV as compared to females (59% vs. 48%), *p* = 0.004. See Table 2. This was consistent with male respondents having higher percentages that had driven/ridden a UTV in the past year and higher proportions that rode at least daily, both *p* < 0.001. However, there were still nearly two-thirds of females (63%) that had driven/ridden a UTV in the past year and half of those did so at least weekly. Although similar proportions of male and female drivers/riders had been on public roads in the past year with UTVs, males had higher proportions reporting more frequent use on unpaved and paved roads (*p* < 0.001 and *p* = 0.022, respectively).

**Table 2:** Bivariate analysis for sex and age regarding UTV ownership and riding among 2024 Iowa FFA Leadership Conference survey participants (*N* = 1,029)

	Sex			Age				
	Female *n* (Col %)^a^	Male *n* (Col %)^a^	*p* value	13–15 yrs *n* (Col %)^a^	16–18 yrs *n* (Col %)^a^			*p* value
Own a UTV								
No Yes	308 (52) 284 (48)	166 (41) 243 (59)	0.004	183 (47) 208 (53)	293 (47) 325 (53)			0.851
Has Driven/Ridden UTV^b^								
No Yes	217 (37) 375 (63)	92 (22) 318 (78)	< 0.001	126 (32) 265 (68)	184 (30) 435 (70)			0.401
How Frequently Driven/Ridden UTV^b^								
Almost Daily About Weekly About Monthly Few Times a Year	93 (25) 92 (25) 77 (21) 113 (30)	119 (37) 87 (27) 48 (15) 64 (20)	< 0.001	77 (29) 70 (26) 51 (19) 67 (25)	138 (32) 112 (26) 74 (17) 111 (26)			0.832
Driven/Ridden UTV on Unpaved Public Roads^b^								
No Yes	25 (7) 346 (93)	15 (5) 300 (95)	0.271	21 (8) 239 (92)	20 (5) 413 (95)			0.062
How Frequently Driven/Ridden UTV on Unpaved Public Road^b^								
Almost Daily About Weekly About Monthly Few Times a Year	81 (23) 84 (24) 82 (24) 99 (29)	103 (34) 84 (28) 42 (14) 71 (24)	< 0.001	64 (27) 64 (27) 49 (21) 62 (26)	123 (30) 104 (25) 77 (19) 109 (26)			0.823
Driven/Ridden UTV on Paved Public Road^b^								
No Yes	48 (13) 323 (87)	38 (12) 277 (88)	0.730	33 (13) 227 (87)	54 (12) 379 (88)			0.932
How Frequently Driven/Ridden UTV on Paved Public Road^b^								
Almost Daily About Weekly About Monthly Few Times a Year	59 (18) 74 (23) 76 (24) 114 (35)	67 (24) 80 (29) 60 (22) 70 (25)	0.022	46 (20) 64 (28) 44 (19) 73 (32)	83 (22) 91 (24) 93 (25) 112 (30)			0.371

^a^The sum of n for a variable may not equal the total Group N due to missing values, and percentages may not add up to 100 due to rounding

^b^Only includes those who had driven/ridden a UTV in past year

Comparisons between younger teens aged 13–15 years and older teens aged 16–18 years were used for bivariate analysis. See Table 2. No meaningful differences were observed between the two age groups regarding UTV ownership, driving/riding UTVs, or their use on unpaved and paved public roads.

Bivariate analyses were also performed for place of residence. See Table 3. Differences were observed across most comparisons except with regard to having driven/ridden on a paved public road in the past year. Generally, respondents that lived on farms had the highest proportions that owned UTVs, had driven/ridden UTVs in the past year, and drove/rode UTVs most frequently in general as well as on public roads. Similarly, those that lived in the country, but not on a farm had greater percentages that owned UTVs, drove/rode UTVs, and did so more frequently in general and on public roads than those respondents who lived in towns. Still, almost a third of participants who lived in a town (31%) owned a UTV with nearly three-fifths (57%) having driven/ridden a UTV in the past year and a third of these (33%) reporting driving/riding at least weekly.

**Table 3:** Bivariate analysis for place of residence regarding UTV ownership and riding among 2024 Iowa FFA Leadership Conference survey participants (*N* = 1,029)

	Farm *n* (Col%)^a^	Country/ not farm *n* (Col%)^a^	In town *n* (Col%)^a^	*p* value
Own Vehicle				
No Yes	161 (32) 342 (68)	107 (52) 98 (48)	208 (69) 93 (31)	< 0.001
Has Driven/Ridden UTV				
No Yes	110 (22) 394 (78)	71 (35) 134 (65)	129 (43) 172 (57)	< 0.001
How Frequently Driven/Ridden UTV^b^				
Almost Daily About Weekly About Monthly Few Times a Year	164 (42) 102 (26) 62 (16) 66 (17)	24 (18) 51 (38) 25 (19) 34 (25)	27 (16) 29 (17) 38 (22) 78 (45)	< 0.001
Driven/Ridden UTV on Unpaved Public Roads^b^				
No Yes	13 (3) 376 (97)	10 (8) 123 (92)	18 (11) 153 (89)	0.003
How Frequently Driven/Ridden UTV on Unpaved Public Road^b^				
Almost Daily About Weekly About Monthly Few Times a Year	143 (38) 96 (26) 69 (18) 68 (18)	23 (19) 42 (34) 27 (22) 31 (25)	21 (14) 30 (20) 30 (20) 72 (47)	< 0.001
Driven/Ridden UTV on Paved Public Road^b^				
No Yes	44 (11) 345 (89)	21 (16) 112 (84)	22 (13) 149 (87)	0.400
How Frequently Driven/Ridden UTV on Paved Public Road^b^				
Almost Daily About Weekly About Monthly Few Times a Year	90 (26) 92 (27) 79 (23) 84 (24)	22 (20) 35 (31) 24 (21) 31 (28)	17 (11) 28 (19) 34 (23) 70 (47)	< 0.001

^a^The sum of n for a variable may not equal the total Group N due to missing values, and percentages may not add up to 100 due to rounding

^b^Only includes those who had driven/ridden a UTV in past year

### UTV safety and design for roadways

Overall, more than three-fifths of respondents believed it was safe to drive UTVs on unpaved (64%) and paved (63%) public roads. See Table 4. Only 7% and 13% of adolescents stated it was unsafe to drive on unpaved and paved roads, respectively. A higher proportion of males thought it was safe or very safe to drive UTVs on unpaved (79% vs. 54%) and paved (75% vs. 52%) public roads as compared to females, both *p* < 0.001. Older teens and those from farms also had greater percentages that believed it was safe to drive UTVs on unpaved public roads, both *p* < 0.01. Those with the highest percentage that stated it was safe to drive on unpaved and paved public roads were individuals that drove/rode at least weekly on unpaved (309/354, 87% believed it safe) and paved (240/283, 85% believed it safe) public roads. Among UTV drivers/riders, higher odds of perceiving UTV use on unpaved and paved public roads as being safe was observed among males, UTV owners and those that had driven on roads.

**Table 4:** Descriptive, bivariate, and logistic regression analyses of how safe rural adolescents believe it is to ride utility task vehicles (UTVs) on unpaved and paved public roads among 2024 Iowa FFA Leadership Conference survey participants (*N* = 1,029)

How safe do you feel it is to drive a UTV on an unpaved public road?^a^							How safe do you feel it is to drive a UTV on a paved public road?^a^					
Very safe			240 (24%)				Very safe			230 (23%)		
Safe			399 (40%)				Safe			410 (40%)		
Neither safe/unsafe			289 (29%)				Neither safe/unsafe			247 (24%)		
Unsafe			45 (5%)				Unsafe			109 (11%)		
Very unsafe			18 (2%)				Very unsafe			21 (2%)		
	Unpaved Public Road						Paved Public Road					
	Bivariate Analysis				Logistic Regression^b^		Bivariate Analysis				Logistic Regression^b^	
	Safe/very safe *n* (Row%)^a^	Neither or unsafe *n* (Row%)^a^		*p* value	aOR	95% CI	Safe/very safe *n* (Row%)^a^	Neither or unsafe *n* (Row%)^a^	*p *value		aOR	95% CI
Sex												
Female Male	317 (54) 316 (79)	265 (46) 85 (21)		< 0.001	1.0 ref		304 (52) 299 (75)	278 (48) 102 (25)	< 0.001		1.0 ref	
					3.56	2.37–5.34					2.76	1.91–3.97
Age												
13–15 16–18	227 (59) 412 (68)	155 (41) 197 (32)		0.008	1.0 ref		225 (59) 385 (63)	157 (41) 224 (37)	0.174		1.0 ref	
					1.29	0.88–1.88					1.14	0.80–1.63
Residence												
Farm Country/not farm Town	347 (70) 130 (65) 162 (55)	149 (30) 71 (35) 132 (45)		< 0.001	1.09 1.00 1.0 ref	0.69–1.73 0.58–1.74	315 (64) 127 (63) 168 (57)	181 (36) 74 (37) 126 (43)	0.179		1.03 1.11 1.0 ref	0.67–1.60 0.65–1.89
Family Owns UTV												
No Yes	223 (48) 415 (79)	240 (52) 112 (21)		< 0.001	1.0 ref		228 (49) 381 (72)	235 (51) 146 (28)	< 0.001		1.0 ref	
					2.65	1.72–4.09					1.55	1.02–2.35
Has Driven/Ridden UTV^b^												
No Yes	131 (44) 508 (73)	168 (56) 184 (27)		< 0.001	Not used in model		131 (44) 479 (69)	168 (56) 213 (31)	< 0.001		Not used in model	
Driven/Ridden UTV Frequency												
Monthly or less At least weekly	178 (60) 330 (84)	121 (40) 63 (16)		< 0.001	Not used in model		171 (57) 308 (78)	128 (43) 85 (22)	< 0.001		Not used in model	
Driven/Ridden UTV on Unpaved Public Roads												
No Yes	14 (34) 494 (76)	27 (66) 156 (24)		< 0.001	1.0 ref 4.54		--- ---	--- ---	---			
						2.17–9.49						
Driven/Ridden UTV on Paved Public Roads												
No Yes	--- ---	--- ---		---			35 (40) 444 (74)	52 (60) 160 (26)	< 0.001		1.0 ref 4.21	
												2.57–6.89

aOR, adjusted odds ratio; CI, confidence interval;

^a^The sum of n for a variable may not equal the total Group N due to missing values, and percentages may not add up to 100 due to rounding

^b^Only includes those who had driven/ridden a UTV in past year and the total number of cases used in the logistic regression model was 682

The majority of respondents also agreed that UTVs are designed for unpaved (63%) and paved (54%) public roads. See Table 5. Just 4% and 9% disagreed and believed that UTVs were not designed for unpaved and paved public roads, respectively. Higher percentages that agreed UTVs were designed for public roads included respondents that were male, lived on farms, owned UTVs, had driven/ridden UTVs, reported more frequent use, or had driven on roads. As compared to younger teens, older teens also had a higher proportion who believed that UTVs were designed for unpaved public roads.

**Table 5:** Descriptive and bivariate analysis of the extent rural adolescents agree or disagree that utility task vehicles (UTVs) are designed for use on unpaved and paved public roads among 2024 Iowa FFA Leadership Conference survey participants (*N* = 1,029)

UTVs are designed for unpaved public road surfaces.^a^					UTVs are designed for paved public road surfaces.^a^			
Strongly agree		233 (24%)			Strongly agree	191 (19%)		
Agree		384 (39%)			Agree	341 (35%)		
Neither agree/disagree		326 (33%)			Neither agree/disagree	359 (36%)		
Disagree		33 (3%)			Disagree	71 (7%)		
Strongly disagree		13 (1%)			Strongly disagree	19 (2%)		
	Unpaved Public Road				Paved Public Road			
	Strongly agree/agree *n* (Row%)^a^		Neither or disagree *n* (Row%)^a^	*p* value	Strongly agree/agree *n* (Row%)^a^		Neither or disagree *n* (Row%)^a^	*p* value
Sex								
Female Male	319 (55) 291 (73)		261 (45) 110 (27)	< 0.001	275 (48) 251 (63)		298 (52) 149 (37)	< 0.001
Age								
13–15 16–18	223 (59) 394 (65)		158 (41) 214 (35)	0.048	200 (53) 332 (55)		177 (47) 272 (45)	0.558
Residence								
Farm Country/not farm Town	337 (68) 123 (62) 157 (54)		159 (32) 77 (39) 136 (46)	< 0.001	288 (59) 106 (53) 138 (48)		204 (41) 93 (47) 152 (52)	0.012
Family Owns UTV								
No Yes	221 (48) 396 (75)		242 (52) 129 (25)	< 0.001	201 (44) 330 (64)		260 (56) 189 (36)	< 0.001
Has Driven/Ridden UTV								
No Yes	127 (43) 490 (71)		171 (57) 201 (29)	< 0.001	116 (39) 416 (61)		180 (61) 269 (39)	< 0.001
Driven/Ridden UTV Frequently^b^								
No Yes	181 (61) 309 (79)		117 (39) 84 (21)	< 0.001	148 (50) 268 (69)		146 (50) 123 (31)	< 0.001
Driven/Ridden UTV on Unpaved /Paved Public Roads^b^								
No Yes	49 (56) 441 (73)		38 (44) 162 (27)	0.001	32 (37) 384 (64)		54 (63) 214 (36)	< 0.001
Driven/Ridden UTV Frequently on Public Roads^b^								
No Yes	208 (65) 233 (82)		112 (35) 50 (18)	< 0.001	170 (54) 214 (76)		147 (46) 67 (24)	< 0.001

^a^The sum of n for a variable may not equal the total Group N due to missing values, and percentages may not add up to 100 due to rounding

^b^Only includes those who had driven/ridden a UTV in past year

Greater than half of the study participants agreed that manufacturers state that UTVs are safe to ride on unpaved (54%) and paved (51%) public roads. See Table 6. Only 7% and 8% disagreed with this statement regarding unpaved and paved roads, respectively. Higher proportions of respondents who were male, UTV owners, UTV drivers/riders, had been on roads, or reported more frequent use agreed manufacturers state that UTVs are safe to ride on unpaved as well as paved public roads.

**Table 6:** Descriptive and bivariate analysis of the extent rural adolescents agree or disagree that utility task vehicle (UTV) manufacturers state UTVs are safe to ride on unpaved and paved public roads among 2024 Iowa FFA Leadership Conference survey participants (*N* = 1,029)

UTV manufacturers state UTVs are safe to ride on unpaved public roads.^a^					UTV manufacturers state UTVs are safe to ride on paved public roads.^a^			
Strongly agree		190 (19%)			Strongly agree	167 (17%)		
Agree		343 (35%)			Agree	337 (34%)		
Neither agree/disagree		378 (39%)			Neither agree/disagree	400 (41%)		
Disagree		59 (6%)			Disagree	54 (6%)		
Strongly disagree		11 (1%)			Strongly disagree	19 (2%)		
	Unpaved Public Road				Paved Public Road			
	Strongly agree/agree *n* (Row%)^a^		Neither or disagree *n* (Row%)^a^	*p* value	Strongly agree/agree *n* (Row%)^a^		Neither or disagree *n* (Row%)^a^	*p* value
Sex								
Female Male	269 (47) 257 (65)		307 (53) 140 (35)	< 0.001	262 (46) 236 (60)		311 (54) 160 (40)	< 0.001
Age								
13–15 16–18	193 (51) 340 (57)		187 (49) 261 (43)	0.077	185 (49) 319 (53)		194 (51) 279 (47)	0.167
Residence								
Farm Country/not farm Town	281 (57) 111 (56) 141 (49)		210 (43) 89 (45) 149 (51)	0.061	270 (55) 94 (47) 140 (48)		217 (45) 106 (53) 150 (52)	0.054
Family Owns UTV								
No Yes	202 (44) 330 (64)		259 (56) 189 (36)	< 0.001	192 (42) 311 (60)		267 (58) 206 (40)	< 0.001
Has Driven/Ridden UTV								
No Yes	127 (43) 406 (59)		170 (57) 278 (41)	< 0.001	117 (40) 387 (57)		178 (60) 295 (43)	< 0.001
Driven/Ridden UTV Frequently^b^								
No Yes	144 (49) 262 (67)		151 (51) 127 (33)	< 0.001	136 (46) 251 (65)		158 (54) 137 (35)	< 0.001
Driven/Ridden UTV on Unpaved /Paved Public Roads^b^								
No Yes	11 (27) 395 (62)		30 (73) 247 (38)	< 0.001	30 (35) 357 (60)		56 (65) 238 (40)	< 0.001
Driven/Ridden UTV Frequently on Public Roads^b^								
No Yes	158 (54) 237 (68)		136 (46) 111 (32)	< 0.001	157 (50) 200 (72)		160 (50) 78 (28)	< 0.001

^a^The sum of n for a variable may not equal the total Group N due to missing values, and percentages may not add up to 100 due to rounding

^b^Only includes those who had driven/ridden a UTV in past year

### UTV/ATV roadway laws

Just over half of respondents (56%, 549/973) were aware of the 2022 Iowa law allowing UTVs/ATVs on public roads in Iowa. Only 18% (177/987) knew an operator had to be 18 years old with a valid driver’s license to drive UTVs/ATVs on public roads in Iowa. The vast majority thought it was younger, with almost three-fifths (59%, 585/987) believing it was 16 years.

## Discussion

About half of study respondents stated their families owned at least one UTV and nearly 70% had driven/ridden a UTV in the past year. Of drivers/riders, UTV usage was high with nearly three-fifths (57%) having ridden UTVs at least weekly. Although there were some observed differences, UTV exposure was high among all groups including by sex, age and residence. Moreover, almost all of the UTV drivers/riders had been on unpaved (94%) and paved (87%) public roads in the past year with a significant proportion doing so at least weekly (51% and 41%, respectively). The majority of respondents agreed it was safe to drive UTVs on both unpaved and paved public roads and believed the falsehoods that; (a) they are designed for public roads and (b) manufacturers state UTVs are safe for use on public roads. Generally, males, UTV owners, UTV riders, those who had ridden on public roads and more frequent riders all had greater proportions that held these misconceptions. In addition, the proportions of adolescents correctly disagreeing with the statements were quite low (4–13%).

Similar to respondents in our study, children and teenagers in many rural areas have high exposure to UTVs [6, 19]. A study of 2,075 Iowa FFA members surveyed in 2015 and 2016 revealed that over four-fifths of them had been on a ROV/UTV, and the vast majority of these had ridden UTVs on public roads even though it may have been illegal in their county at the time [19]. One concerning trend comparing the results of the 2015/2016 survey of Iowa FFA members with this 2024 survey is that the proportion reporting riding on paved public roads in the past year increased from 77% to 87% and the proportion reporting at least weekly riding on paved public roads went from 34% to 41%.

Although there are a few youth UTV models for children 10 years and older, almost all UTVs are recommended for operators ≥ 16 years of age. In addition, manufacturers state a person should only be a passenger in a UTV if they can sit with their back against the seat with their feet flat on the floor, often this is when they are at least 12 years old. Targeted education of rural residents regarding the safe use of UTVs, and in particular the dangers of riding UTVs on public roads and ways to reduce that risk, is vitally needed.

A previous study of adult attendees of the 2022 Farm Progress Show showed patterns similar to those observed in our study [17]. UTV usage was high (70%) with nearly two-thirds of these riding UTVs at least weekly. Road use was also similar to our study, with 98% of adult drivers/riders reporting being on unpaved roads and 89% on paved roads in the past year. Over three-fifths of the Farm Progress Show attendees believed UTVs were safe for use on unpaved and paved public roads. The majority also agreed that UTVs were designed for unpaved public roads (64%) and that manufacturers state they are safe to drive on unpaved public roads (54%). Just less than half agreed UTVs were designed for paved public roads and that manufacturers state they are safe for paved public roads (both 46%), slightly lower than in our study where over half of teenagers agreed with these statements. Farm Progress Show adults correctly disagreeing with the statements was higher than our study adolescents but still concerningly low (8–26%).

The misconceptions regarding UTV operation on public roads held by many adolescents in our study and among Farm Progress Show attendees suggest potential gaps in the communication of safety information to consumers. These erroneous beliefs may reflect an inadequate emphasis by manufacturers and powersport dealers regarding the off-road design of UTVs and the associated risks of public roadway operation. Addressing this gap may require standardized safety counseling at the point of sale and a more prominent focus on the hazards of public roadway use in manufacturer media and product advertising.

Current public misperceptions may be influenced by legislative actions allowing UTVs on public roads–despite the vehicle’s non-compliance with Federal Motor Vehicle Safety Standards. Such ordinances are likely perceived by the public as an indication that roadway use is safe. It is essential that the UTV industry not only increase consumer outreach but also engage more actively in educating government officials on the safety risks of UTVs on public roads. Furthermore, UTV manufacturers should prioritize proactive advocacy to ensure legislation aligns with engineering parameters and the safe use of their products to improve public safety outcomes.

### Limitations

Our study was conducted in a single Midwestern state with the vast majority being Non-Hispanic White individuals, so our findings may not be generalizable to other states or communities with more racial/ethnic diversity. In addition, the survey respondents were a convenience sample of primarily rural teenagers who were FFA members attending a state conference, so they may not be representative of the entire state, especially more urban areas. Thus, the study results are best interpreted as an exploration of associations within the study sample rather than making population-level inferences. Despite this, nearly all 99 counties in the state were represented by study participants. Responses were self-reported and may have been affected by recall bias and social desirability bias. However, surveys were completed independently and anonymously with the intention of decreasing this social desirability effect. Future research using representative samples would be valuable to further evaluate these patterns at a population level.

## Conclusions

A substantial majority of study participants reported UTV operation on public roads, with most perceiving the practice as safe on both paved and unpaved roadway surfaces. Respondents also had a high prevalence of misconceptions regarding UTV design specifications and manufacturer safety guidelines. Our findings suggests knowledge regarding the hazards of UTV operation on public roadways is limited and highlights a clear opportunity for enhanced education of UTV consumers and of the general public. Furthermore, vehicle legislation should prioritize congruence with engineering parameters and safe use—specifically the intended off-road use only design of ATVs and UTVs. Finally, the powersports industry could play a vital role in addressing current knowledge gaps and improving safety outcomes by greatly expanding their efforts in communicating the risks of using off-road vehicles on public roadways.

## Supplementary Information

Supplementary Material 1

## Data Availability

Data and materials are available to other parties for research purposes after a data sharing agreement plan is agreed to and signed. Those interested should contact the corresponding author.

## Declarations

### About this supplement

This article has been published as part of Injury Epidemiology Volume 13 Supplement 1, 2026: Proceedings of the 30th Annual Injury Free Coalition for Kids^®^ Conference: Forging New Frontiers: injury Prevention in Evolving Environments. The full contents of the supplement are available online at https://link.springer.com/journal/40621/volumes-and-issues/13-1/supplement.

## Abbreviations

ATV: All-terrain vehicle
FFA: Formerly Future Farmers of America
ORV: Off-road vehicle
ROV: Recreational off-highway vehicle
ROPS: Roll-over protective structure
SFCH: Stead Family Children’s Hospital
U.S.: United States
UTV: Utility task vehicle
