# Supplementary material for: Rural iowa adolescents’ use, knowledge and attitudes regarding UTVs on public roads
Source: Inj Epidemiol. 2026 Oct 28;13(Suppl 1):68. doi: 10.1186/s40621-026-00707-z (PMC13717961; doi:10.1186/s40621-026-00707-z)
Supplement: Supplementary file 1 [file 40621_2026_707_MOESM1_ESM.docx]

**2024 Iowa FFA Leadership Conference UTVs on Roadways Survey, April 15-16, 2024**

**Section 1: Who are you?**

1. What is your sex?	Female	Male	Non-Binary	Other ___________

2. How old are you?	______________ years old

3. Where do you live?	On a farm	In the country, not on a farm	In town

4. What do you consider your race/ethnicity?

White/Caucasian	Black/African American	Hispanic or Latinx	Asian	Other/Mixed

**Section 2: UTV Use.**

**IMPORTANT--PLEASE READ BEFORE CONTINUING**:

A utility task vehicle (UTV) is as pictured here and has a steering wheel, foot pedals for braking and acceleration, bench or side-by-side seating with seat belts or harnesses, and a roll-over protective structure.

An unpaved public road includes gravel or dirt. A paved public road includes concrete, chip and seal, or tar.

13. Do you or your family own a **UTV**? …………………………………………………	Yes	No

a

14. How often do you drive/ride a UTV?

Almost Daily	About Weekly	About Monthly	Few Times a Year or Less	Never

a

15. How often do you drive/ride a UTV on an unpaved public road?

Almost Daily	About Weekly	About Monthly	Few Times a Year or Less	Never

a

16. How often do you drive/ride a UTV on a paved public road?

Almost Daily	About Weekly	About Monthly	Few Times a Year or Less	Never

a

**Section 3: What Do You Think Regarding UTVs?**

17. How safe do you feel it is to drive a UTV on an unpaved public road?

Very Safe	Safe	Neither safe or unsafe	Unsafe	Very Unsafe

18. How safe do you feel it is to drive a UTV on a paved public road?

Very Safe	Safe	Neither safe or unsafe	Unsafe	Very Unsafe

19. Please indicate how much you agree or disagree with each of the following statements:

a. UTVs are designed for unpaved public road surfaces.

Strongly Agree	Agree	Neither agree or disagree	Disagree	Strongly Disagree

b. UTVs are designed for paved public road surfaces.

Strongly Agree	Agree	Neither agree or disagree	Disagree	Strongly Disagree

c. Most UTV deaths occur on public roads.

Strongly Agree	Agree	Neither agree or disagree	Disagree	Strongly Disagree

d. UTV manufacturers state UTVs are safe to ride on unpaved public roads.

Strongly Agree	Agree	Neither agree or disagree	Disagree	Strongly Disagree

e. UTV manufacturers state UTVs are safe to ride on paved public roads.

Strongly Agree	Agree	Neither agree or disagree	Disagree	Strongly Disagree

20. Were you aware that the Iowa Legislature passed a law in 2022 allowing ATVs and UTVs on all unpaved county roads and with some restrictions on two-lane state and county paved highways?	Yes	No

21. If you have a valid driver’s license, how old do you need to be to drive ATVs and UTVs on county roads?

14 years	15 years	16 years	17 years	18 years

**Thank You For Helping with this Survey!**
